# Direct oral anticoagulant rivaroxaban for secondary prevention of catheter-related thrombosis in children with intestinal failure: real-life data

**DOI:** 10.1016/j.rpth.2025.103231

**Published:** 2025-10-22

**Authors:** Johannes Hilberath, Antje Ballauff, Aline Rückel, Ludger Sieverding, Drieke Vermeulen, Wolfgang Eberl, Steffen Hartleif, Justus Lieber, Jörg Michel, Ekkehard Sturm, Johannes Nordmeyer, Vanya Icheva

**Affiliations:** 1 Paediatric Gastroenterology and Hepatology, Department of Haematology and Oncology, University Children’s Hospital Tübingen, Tübingen, Germany; 2 Department of Pediatrics, Helios Children’s Hospital, Krefeld, Germany; 3 Department of Pediatrics and Adolescent Medicine, University Hospital Erlangen, Friedrich-Alexander-Universität (FAU) Erlangen-Nürnberg, Germany; 4 Department of Pediatric Cardiology, Pulmonology and Intensive Care Medicine, University Children’s Hospital Tübingen, Tübingen, Germany; 5 Department of Pediatrics, Klinikum Braunschweig, Braunschweig, Germany; 6 Department of Pediatric Surgery and Urology, University Children’s Hospital, Tübingen, Germany

**Keywords:** factor Xa inhibitors, intestinal failure, parenteral nutrition, rivaroxaban, venous thrombosis

## Abstract

**Background:**

Catheter-related thrombosis (CRT) affects 20% to 57% of pediatric patients with intestinal failure (IF) on long-term parenteral nutrition with risk of venous access loss. Subcutaneous low-molecular-weight heparin is burdensome, with 35% of long-term treated children experiencing recurrent thrombosis. Data on the direct oral anticoagulant (DOAC) rivaroxaban in patients with IF are limited.

**Objectives:**

This study aimed to evaluate the characteristics and outcomes of children with IF receiving rivaroxaban for secondary prevention of CRT.

**Methods:**

We conducted a multicenter, retrospective longitudinal cohort study in children (0-17 years) with IF treated with rivaroxaban between July 2021 and May 2025. Clinical, diagnostic, imaging, and laboratory data were collected from medical records and analyzed. The study was approved by the institutional ethics board (709/2023BO2).

**Results:**

The study included 26 children; 15 children switched to anticoagulation with rivaroxaban to improve adherence due to incompliance with subcutaneous application. Median follow-up until the last imaging assessment was 436 days. In 11 children (42%), rivaroxaban dosage was adjusted due to insufficient drug levels. Two patients demonstrated CRT, corresponding to a rethrombosis rate of 7.7% or 0.2/1000 catheter days. Four patients (15%) experienced intestinal bleeding.

**Conclusion:**

This is the first study to provide real-life insights into the use of the DOAC rivaroxaban in children with intestinal failure, demonstrating its potential for secondary CRT prevention and child-friendly administration with very low rethrombosis rate. However, dose adjustments based on therapeutic drug monitoring might be necessary due to heterogeneous variability in absorption. Future prospective and comparative trials are needed to confirm these findings.

## Essentials

• Children with intestinal failure on parenteral nutrition risk catheter-related thrombosis (CRT).
• Study explores oral rivaroxaban for prevention of CRT in children with intestinal failure.
• Analysis demonstrates rivaroxaban’s potential: feasibility, adherence, safety, and low rethrombosis.
• Study highlights need of drug monitoring and dose adjustments due to variable drug absorption.

## 1 Introduction

Pediatric chronic intestinal failure (IF) is defined as a critical reduction of gut mass or function below the minimum required for maintaining nutritional and/or fluid homeostasis [1]. Treatment involves providing home-parenteral nutrition (PN) via a long-term central venous catheter (CVC) to support survival.

However, PN use is associated with catheter-related complications such as venous thromboembolism including catheter-related thrombosis (CRT) and loss of venous access sites [2,3]. CRT affects 20% to 57% of pediatric patients with IF on long-term PN [4,5]. In an international survey among European IF centres, 46% of the teams responded to use prophylactic anticoagulation [6]. Among children with IF on long-term anticoagulation prophylaxis with subcutaneous low-molecular-weight heparin (LMWH), 35% of developed secondary thrombosis [7]. The American College of Chest Physicians’ 2012 guidelines recommend prophylactic anticoagulation for pediatric patients with long-term home-PN and for children with CVC for secondary prophylaxis until catheter removal [8].

However, practices for anticoagulation management vary among pediatric patients with IF and potentially lifelong PN [9]. Subcutaneous LMWH administration is troublesome for most pediatric patients. Vitamin K antagonist treatment can be challenging due to hepatopathy and bleeding risk. As many centers continue anticoagulation until CVC removal [9], the availability of a safe and child-friendly anticoagulation is needed. Direct oral anticoagulants (DOACs) such as rivaroxaban offer an oral formulation and could meet this demand. Rivaroxaban, a reversible direct factor Xa inhibitor is absorbed in the upper gastrointestinal tract with high bioavailability and is approved for venous thromboembolism treatment and secondary prophylaxis in patients from birth to 18 years [10]. Notably, rivaroxaban does not require dietary adjustments, unlike vitamin K antagonists. However, pharmacotherapy in patients with intestinal malfunction such as short bowel syndrome remains challenging and literature on oral medication use in this population is scarce [11]. In addition, despite the common use of anticoagulation in pediatric patients with IF to prevent CRT, data on DOACs in children with IF is not available [12], except for 1 case report [13]. The recent review by Jaffray and Young [14] on DOAC use in children suggests future studies should focus on CRT prevention. Our study aimed to evaluate the feasibility, safety, and efficacy of rivaroxaban for secondary prevention of CRT in pediatric patients with IF, addressing the gap in data for DOAC use in this vulnerable population.

## 2 Methods

This multicenter, retrospective longitudinal cohort study included children (0-17 years) with chronic IF and PN via a long-term venous catheter who received rivaroxaban for secondary prevention of CRT between July 2021 and May 2025. Patients without a CVC were excluded. Three national hospitals experienced in the management of children with chronic IF contributed to this study.

Clinical, diagnostic, imaging, and laboratory data were collected from medical records, including patient sex, age, body weight, underlying IF etiology, and residual bowel anatomy. Ethnic group information was collected to describe the study cohort. The anticoagulative medications before rivaroxaban and reasons for switch to rivaroxaban were assessed. Initially, body weight-adjusted dosing for rivaroxaban followed manufacturer’s instructions. Peak plasma drug level was measured 3 to 4 hours after rivaroxaban intake, trough level before next dose administration. Plasma target values were based on the EINSTEIN Junior study data for age-dependent steady-state concentrations described in the product information in the context of the European Medicines Agency approval (https://www.ema.europa.eu/en/documents/product-information/xarelto-epar-product-information_en.pdf) (Table 1) [15]. Dose increases were documented if due to weight gain or drug level outside targeted range. Outcome parameters included recurrent thrombosis, bleeding complications, side effects, drug discontinuation, and mortality. Two different points were analyzed for the assessment of rethrombosis: last clinical evaluation of catheter patency and vascular imaging by ultrasound or computed tomography. CRT occurrence was assessed through routine ultrasound surveillance or imaging when clinically suspected. Ultrasonography was performed by a trained radiologist or cardiologist.

**Table 1:** Summary statistics (geometric mean [90% interval]) of rivaroxaban peak plasma concentrations (μg/L) by dosing regimen and age (as provided in the product information) [15].

Dosing regimen	Age (y)				
	12 to <18	6 to <12	2 to <6	0.5 to <2	Birth to <0.5
Once daily	241.5 (105-484)	229.7 (91.5-777)	—	—	—
Twice daily	—	145.4 (46.0-343)	171.8 (70.7-438)	—	—
Thrice daily	—	—	164.7 (108-283)	114.3 (22.9-346)	108.0 (19.2-320)

Descriptive analyses were performed using IBM SPSS Statistics, version 28.0. Statistical group comparison included the Mann–Whitney *U* test for nonnormally distributed variables or the chi-squares test for categorical variables. Children requiring rivaroxaban dose increase due to low drug levels were compared with those without increase during follow-up. Violin plots for drug levels were created with GraphPad Prism version 10.1.1 for Windows (GraphPad Software; www.graphpad.com). This study followed the ethical principles of Declaration of Helsinki and was approved by the local institutional ethics committee (709/2023BO2).

## 3 Results

### 3.1 Patient characteristics

Overall, 26 pediatric patients with IF, long-term CVC, and indication for secondary CRT prophylaxis were enrolled (Table 2): median age at start of rivaroxaban was 88.5 months, median weight was 22 kg, and 58% of patients were girls. Short bowel syndrome was the underlying IF etiology in 89% of patients.

**Table 2:** Patient characteristics.

Characteristic	Value
Central venous long-term catheter for parenteral nutrition	26 (100)
Age at start of rivaroxaban (mo)	88.5 (3-199; 37-132)
Body weight at start of rivaroxaban (kg)^a^	22 (4.7-67; 11.95-30.2)
Ethnic group	
White	21 (80.8)
Black	0 (0)
Asian	1 (3.8)
Other (including Middle Eastern/North African)	4 (15.4)
Previous treatment with enoxaparin	24 (92.3)
Thrombophilia screening	15 (57.7)
Pathological test result	4/15 (26.7)
No. of previous central venous long-term catheters^b^	4 (1-13; 2-7)
Intestinal failure etiology	
Short bowel syndrome	23 (88.5)
Motility disorder	2 (7.7)
Mucosal enteropathy	1 (3.8)
Intestinal anatomy	
Residual short bowel length from the ligament of Treitz (cm)^c^	30 (0-140; 20-47.5)
Presence of small intestinal stoma	8 (30.8)
Follow-up time from start of rivaroxaban	
Until last clinical assessment (d)	632 (302-928)
Until last ultrasound vascular imaging (d)^d^	436 (173-817.5)

Values are n (%), median (range; IQR), or median (IQR).

a Data missing, *n* = 1.

b Data missing, *n* = 1.

c 2 patients with no resection excluded from this calculation.

d Data missing, *n* = 5.

All but 2 patients received enoxaparin before rivaroxaban treatment. One patient with short bowel syndrome and a residual small bowel length of 15 cm switched from phenprocoumon to rivaroxaban because of unstable international normalized ratio measurements. In another child with short bowel syndrome and 10 cm of small bowel remaining, rivaroxaban was the first-line anticoagulant as preferred by the family. In 15 cases, the indication for switching from enoxaparin to rivaroxaban (15/24, 62.5%) was lack of compliance due to intolerance to subcutaneous application. No concomitant anticoagulants were used. The median follow-up period until the last clinical assessment was 20.5 months (IQR, 9-30 months).

### 3.2 Therapeutic drug monitoring of rivaroxaban

In total, 162 plasma drug level measurements were analyzed (peak, *n* = 121; trough, *n* = 41; median per patient 5.5 [IQR, 3-9]). A total of 82.6% of all measured peak levels were within the targeted range (Figure 1). The median trough levels were 32 μg/L (IQR, 22-47 μg/L) and the median peak levels 96 μg/L (IQR, 57-142 μg/L) for the entire cohort. During follow-up, in 3 children (11.5%), the dosage was adapted due to increased body weight, and in 11 children (42.3%), the rivaroxaban dosage had to be increased because of failure to reach the targeted drug levels. Among this group, 1 patient had a motility disorder leading to a proximal intestinal stoma placement 20 cm after the duodenojejunal flexure. All other patients had short bowel syndrome. The median residual small bowel length was 40 cm (range, 0-100 cm; IQR, 20-70 cm), and small bowel stoma was present in 4 patients. Notably, in 1 patient with 10 cm of small bowel after the ligament of Treitz, the targeted peak drug levels could not be reached despite dose increase of up to 60% above recommended, this patient returned to enoxaparin after 1 month of anticoagulation with rivaroxaban. In 2 patients with IF (7.8%), a dose reduction was performed: 1 patient with short bowel syndrome and 5 cm of residual small bowel length due to high drug level, and 1 patient with mucosal enteropathy following an episode of clinically relevant intestinal bleeding. There was no significant difference between children with an increase in dosage due to low drug levels and those with no increase during follow-up, including IF etiology, residual small bowel length, presence of intestinal stoma, concomitant treatment with teduglutide, and time of follow-up (Table 3).

**Figure:**
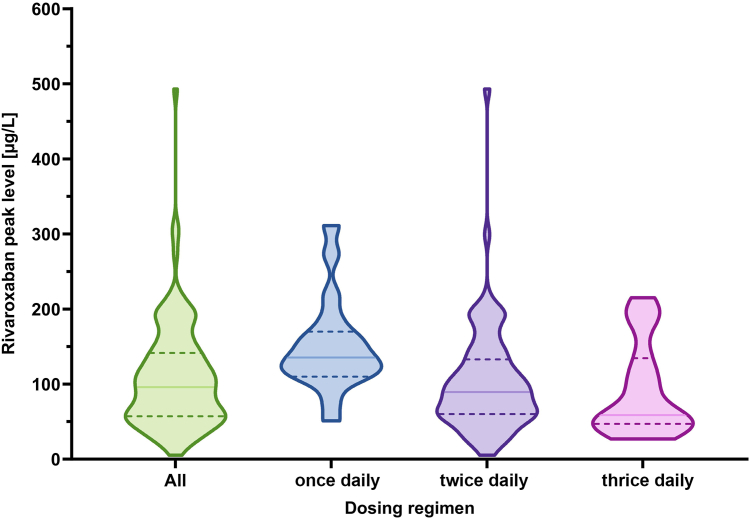
Rivaroxaban drug monitoring: violin plot showing peak levels in all patients (all) and grouped by dosing regimen (once daily, twice daily, and thrice daily) with median (solid lines) and IQR (dashed lines), and frequency distribution of the data. For age- and regimen-dependent steady-state drug concentration ranges, see Table 1.

**Table 3:** Comparison of patients with rivaroxaban dosage increase due to low drug levels vs no dosage increase during follow-up.

Characteristic	Dosage increase (*n* = 11 patients)	No dosage increase (*n* = 15 patients)	*P*
Age at start of rivaroxaban (mo)	102 (5-162; 26-144)	75 (3-199; 38-132)	.959^a^
Body weight at start of rivaroxaban (kg)^b^	24.3 (4.95-45.7; 12.1-37), *n* = 10	22.0 (4.7-67.0; 11.8-30)	.683^a^
Intestinal failure etiology			
SBS	10 (90.9)	13 (86.7)	.859^c^
*n*/*n* (%) of all patients with SBS	10/23 (43.5)	13/23 (56.5)	.376^c^
*n*/*n* (%) of all patients in the cohort	10/26 (38.5)	13/26 (50.0)	.402^c^
MD	1 (9.1)	1 (6.7)	.819^c^
*n*/*n* (%) of all patients with MD	1/2 (50)	1/2 (50)	1.0^c^
*n*/*n* (%) of all patients in the cohort	1/26 (3.8)	1/26 (3.8)	1.0c
ME	0 (0)	1 (6.7)	.382^c^
*n*/*n* (%) of all patients with ME	0/1 (0)	1/1 (100)	.157^c^
*n*/*n* (%) of all patients in the cohort	0/26 (0)	1/26 (3.8)	.313^c^
Concomitant treatment with teduglutide	3 (27.3)	1 (6.7)	.150^c^
Intestinal anatomy			
Residual short bowel length from the ligament of Treitz in short bowel patients (cm), *n* = 24	40 (0-100; 20-70), *n* = 11	25 (5-140; 17.5-40), *n* = 13	.228^a^
Presence of small intestinal stoma	4 (36.4)	4 (26.7)	.597^a^
Follow-up time from start of rivaroxaban			
Until last clinical assessment (d)	499 (317-764)	650 (209-928)	.979^a^
Until last ultrasound vascular imaging (d)^d^	382 (175-756), *n* = 9	588 (155-901), *n* = 12	.602^a^

MD, motility disorder; ME, mucosal enteropathy; SBS, short bowel syndrome.

Values are presented as median (range; IQR) or median IQR unless specified.

a Mann-Whitney *U* test.

b Chi-squared test for categorical variables.

c Data missing, *n* = 1.

d Data missing, *n* = 5.

### 3.3 Recurrent venous thrombosis

The median follow-up period until the last vascular imaging was 16 months (IQR, 8-27 months). During this period, 2 patients (7.7%) were classified as having a CRT, corresponding to a rethrombosis rate of 0.2 per 1000 catheter days in the total cohort (Table 4): 1 patient with known factor V Leiden and protein S deficiency demonstrated secondary asymptomatic thrombosis on echocardiographic imaging. No embolism or thrombosis progression occurred. This event was not associated with a prior central line–related infection and no change in anticoagulation therapy was made. Another patient with a known postthrombotic superior vena cava syndrome and increased venous pressure developed impaired lymphatic drainage, leading to chylothorax. Computed tomography angiography confirmed no progression or additional thrombi, and symptoms resolved after interventional occlusion of the thoracic duct. However, rivaroxaban was insufficient in preventing progression of postthrombotic syndrome. Based on a case-by-case decision of the attending physicians, this patient was switched to a vitamin K antagonist after 12 months of treatment with rivaroxaban. No catheter was replaced because of CRT during the follow-up period.

**Table 4:** Overview of adverse events and surgical procedures.

Intestinal bleeding								
No.	Bleeding site	Age at time of event (mo)	IF etiology	Onset after start of rivaroxaban (mo)	Rivaroxaban drug level prior to event (μg/L) and dosing regimen	Platelets prior to event (1/μL)	Gastrointestinal comorbidity	Outcome
1	Proximal intestinal stoma bleeding	46	SBS	8	95 (peak, within targeted range); thrice daily	305,000	Yes (gastritis and duodenitis)	Iron deficiency, no RBC transfusion; rivaroxaban pause and uneventful resume with reduced dosage
2	Intestinal bleeding	121	SBS	0	130 (trough; above targeted range); twice daily	170,000	Yes (small bowel inflammation, erosions and ulcers)	Hb drop and iron deficiency, no RBC transfusion; stop of rivaroxaban
3	Intestinal bleeding	188	ME	31	126 (peak, within targeted range); once daily	238,000	Yes (ileitis)	Hb drop (6.7 mg/dL), 3 RBC transfusions; rivaroxaban pause and uneventful resume with reduced dosage
4	Proximal intestinal stoma bleeding	164	MD	21	193 (peak, within targeted range); twice daily	350,000	Yes (ileitis)	Hb drop (6.7 mg/dL), 1 RBC transfusion; stop of rivaroxaban

Recurrent venous thrombosis								
No.	Type of thrombosis	Age at time of event (mo)	IF etiology	Onset after start of rivaroxaban (mo)	Rivaroxaban drug level prior to event (μg/L) and dosing regimen	CVC location	Thrombophilia	Outcome
1	Thrombus adherent to catheter tip	108	SBS	33	94 (peak, within targeted range); twice daily	IJV (left)	Yes (factor V Leiden and protein S deficiency)	Asymptomatic, continuation of rivaroxaban, no progression; imaging findings unchanged 8 mo later
2	Progression of postthrombotic syndrome	79	SBS	12	199 (peak, within in targeted range); twice daily	IJV (left)	No (thrombophilia screening negative)	Switch from rivaroxaban to vitamin K antagonist

Surgical procedures						
No.	Type of surgery	Age at surgery (mo)]	IF etiology	Surgery after start of rivaroxaban (mo)]	Management prior to surgery	Complications including bleeding events
1	CVC replacement	129	SBS	5	24 h pause of rivaroxaban	No
2	CVC removal	103	SBS	31	48 h pause of rivaroxaban	No
3	CVC replacement	79	SBS	12	24 h pause of rivaroxaban	No
4	Relaparotomy with intestinal resection	4	SBS	0	72 h pause of rivaroxaban	No
5	Bilateral cochlear implantation	10	SBS	5	48 h pause of rivaroxaban	No
	Closure of intestinal stoma	16	SBS	11	48 h pause of rivaroxaban	No
6	Closure of intestinal stoma	25	SBS	20	Pause of rivaroxaban (time span not known)	No
7	Relaparotomy (3×)	131/139/140	SBS	5/14/14	48 h pause of rivaroxaban	No
8	CVC replacement	175	ME	17	48 h pause of rivaroxaban	No
9	Cerebral shunt revision (7×)	6/6/6/7/11/11/19	SBS	0/0/1/1/6/6/14	24 h pause of rivaroxaban	No
10	CVC replacement	ND	SBS	ND	24-48 h pause of rivaroxaban	No
11	CVC replacement (2x)	148/150	MD	4/6	72 h pause of rivaroxaban/48 h pause of rivaroxaban	No
12	Relaparotomy	138	SBS	6	Pause of rivaroxaban (time span not known)	No

Surgical CVC interventions were not due to catheter-related thrombosis.

CVC, central venous catheter; IJV, internal jugular vein; MD, motility disorder; ME, mucosal enteropathy; ND, no data; RBC, red blood cell concentrate; SBS, short bowel syndrome.

### 3.4 Adverse events

During follow-up, 4 patients (15.4%) experienced bleeding events under therapy with rivaroxaban (Table 4). None of these patients had any other known bleeding disorder. In 3 patients, peak drug levels were determined and within targeted range. Among them, 1 patient received a higher dosage of rivaroxaban than recommended for the body weight at the time of bleeding event, based on previous dose increases due to low drug levels. In the other individual, only the trough level was measured and was above targeted range. In 2 of the patients, rivaroxaban was only temporarily discontinued and could be resumed without further complications with a lower dosage regimen. The medication was permanently discontinued in the other 2 patients. Time point of bleeding event ranged from 4 days to 2 years after initiation of rivaroxaban. Bleeding sites were from gastrointestinal tract in all 4 cases and in combination with inflammation, which was pre-existent before the initiation of anticoagulation with rivaroxaban. No other adverse events were observed. One patient (3.8%) died of pre-existing chronic liver failure, unrelated to rivaroxaban. None of the patients were considered for intestinal transplantation because of the loss of vascular access.

### 3.5 Surgery

Twelve patients (46.2%) underwent 22 surgical interventions, and rivaroxaban was stopped up to 72 hours prior, without an anticoagulation bridging strategy (Table 4). No perioperative bleeding complications were observed. Surgeries included laparotomies, cochlear implant surgery, and interventional and surgical CVC exchange in case of catheter-associated infections. No CVC had to be removed or replaced due to CRT.

## 4 Discussion

CRT is a serious and prognosis-limiting complication in children with IF. Venous thrombi increase the risk of embolism, catheter-associated infections, postthrombotic syndrome, and progressive loss of vascular access sites. The critical limitation of access sites for CVC placement is an increasingly common indication for intestinal transplantation [2,9]. Therefore, the prevention of recurrent CRT is of vital importance [16].

### 4.1 Recurrent venous thrombosis

In children with chronic IF, data on CRT are limited, especially for recurrent thrombosis. A retrospective study by Keefe et al. [4] reported an incidence rate of 0.32 venous thromboembolisms per 1.000 catheter days in patients with IF below 21 years of age without prophylactic anticoagulation [4].

In our cohort, only 2 of 26 patients (7.7%) experienced rethrombosis, corresponding to a low recurrence rate of 0.2 per 1000 catheter days. In a study by Schmidt et al. [7], rethrombosis occurred in 9 of 26 (35%) children with IF who were on long-term prophylaxis with LMWH and underwent routine annual vascular ultrasound within a median follow-up period of 531.5 days. Notably, the median follow-up time for clinical assessment in our study was 632 days and that for vascular imaging was 436 days. The lower rethrombosis rate observed in our cohort suggest that the anticoagulation regimen with rivaroxaban was effective in preventing recurrent CRT events in children with IF. The comparable follow-up periods strengthens the validity of our findings. However, further research is needed to directly compare the efficacy of rivaroxaban and LMWH in preventing rethrombosis in children with IF.

### 4.2 Rivaroxaban and therapeutic drug monitoring

Our study is the first case series to longitudinally examine the real-life use of the DOAC rivaroxaban in pediatric patients with IF for the secondary prevention of CRT. To date, only a single case report that showed the successful administration of drug level–adjusted rivaroxaban in an infant with short bowel and congenital nephrotic syndromes has been published [13]. However, the residual short bowel length was not documented in this patient.

Rivaroxaban is mainly absorbed in the proximal gastrointestinal tract (eg, stomach and proximal small intestine) and, therefore, might be a suitable option for children with IF, especially those with short bowel syndrome [12]. Notably, rivaroxaban dosage had to be escalated in 42% of patients to achieve targeted peak levels, underscoring the importance of therapeutic drug monitoring in this high-risk group. Subsequent dose adjustments may be essential both for the effective prevention of recurrent thrombosis and to identify patients with drug absorption failure, as observed in 1 patient in our cohort. In such cases, the necessity for alternative strategies, such as parenteral anticoagulants, should be discussed early. However, overall baseline characteristics, including residual small bowel length, underlying IF pathology and concomitant medication did not influence drug level outliers and the need for a dosage increase significantly. In line with the findings from the EINSTEIN Junior studies, infants and small children in our cohort receiving rivaroxaban 3 times daily exhibit lower peak plasma levels than those receiving once- or twice-daily regimens [17]. This reflects age-dependent pharmacokinetics and the dosing strategy designed to maintain stable drug concentrations but avoid high peaks [18]. Importantly, in our cohort, we could not link the weight-dependent dosing regimen to an increased complication risk. However, the extent to which deviations of the drug levels can be tolerated without dose adjustment needs to be further prospectively investigated using uniform protocols, as optimal dosing strategies for children with IF are unknown.

The majority of patients in our cohort were switched to rivaroxaban because of the drawback of subcutaneous administration or to increase patients’ and families’ adherence to anticoagulation prophylaxis. Notably, no patient discontinued rivaroxaban because of challenging administration, highlighting its child-friendly oral formulation (including liquid). This is of great importance, especially for children with IF and long-term PN, where prophylactic anticoagulation may be continued lifelong or until CVC removal [9].

In general, laboratory monitoring of anticoagulant activity is not required in healthy children. Nevertheless, as shown in our study, frequent drug monitoring could be relevant in children with IF, and the necessary venipuncture adds to this disadvantage. However, in our cohort, plasma drug levels could not be associated to clinical complications such as bleeding or thrombosis.

### 4.3 Bleeding

We observed a relevant rate of gastrointestinal bleeding (15%) during the follow-up period. Two of these 4 patients had to stop rivaroxaban because of significant blood loss, while the other 2 paused and resumed rivaroxaban without further bleeding events, in a reduced-dose regimen. Compared with established anticoagulants, for example, LWMH and vitamin K antagonists, rivaroxaban showed a comparable rate of gastrointestinal bleeding events in the pediatric population [19]. Notably, all patients had a known history of gastrointestinal inflammation as a common, clinically relevant risk factor. Therefore, we recommend the cautious use of rivaroxaban in patients with IF and a known gastrointestinal bleeding tendency due to inflammation, erosions, or ulcers. As patients with IF are at risk of intestinal inflammation [20], it may be beneficial to assess for (occult) blood loss, inflammatory markers, and, if indicated, perform endoscopy as part of the pretreatment workup. In addition, in affected patients, initiating rivaroxaban treatment at a lower dose with early monitoring of peak and trough levels may be advantageous. Notably, in our retrospective study, no other adverse events were recorded, and no patient discontinued rivaroxaban because of side effects other than bleeding.

### 4.4 Surgery

Twelve patients in our cohort underwent surgery during follow-up, while anticoagulation with rivaroxaban was held. It is important to note that there are no consensus guidelines for balancing the risks of bleeding and thrombosis in children [21]. Importantly, no perioperative bleeding complications were observed in our cohort.

### 4.5 Limitations

The limitations of our study are its retrospective design and reliance on patient file documentation. Although the sample size was small, this case series offers the first data on rivaroxaban use in children with this rare disease. Another limitation is the lack of long-term follow-up beyond the median observation periods of 632 days for clinical assessment and 436 days for vascular imaging. While the observation period is comparable with that analyzed by Schmidt et al. [7], further studies with longer follow-up are needed to assess for late rethrombosis events in this population. Vascular imaging for the detection of recurrent CRT was mainly based on ultrasonography of the central veins, which is noninvasive and readily available; however, it could have underestimated the true incidence of CRT in our study. Venography, computed tomography, and magnetic resonance imaging are more accurate mapping tools for the central venous system [12]; however, these imaging modalities were not part of the screening routine at the 3 centers contributing to this study. It should be emphasized that, in addition to unremarkable sonography, there was no clinical suspicion of rethrombosis.

## 5 Conclusion

In conclusion, this is the first study to provide real-life insights into the use of DOACs in children with IF, demonstrating its feasibility, overall safety, and potential for secondary CRT prevention and child-friendly administration. Given the heterogeneous variability in absorption, it might be beneficial to individualize and optimize treatment by dose adjustments based on therapeutic drug monitoring. Careful patient selection and dosing regimen are necessary in cases of intestinal inflammation in order to avoid bleeding complications. Further comparative studies with larger sample sizes and extended follow-up periods are needed to confirm our findings and to investigate uniform treatment protocols based on serum level measurements for optimal dosing in children with IF.

## References

[1] Norsa L., Goulet O., Alberti D., DeKooning B., Domellöf M., Haiden N. (2023). Nutrition and intestinal rehabilitation of children with short bowel syndrome: a position paper of the ESPGHAN Committee on Nutrition. Part 1: from intestinal resection to home discharge. J Pediatr Gastroenterol Nutr.

[2] Smith R.W., Pettini M., Gulden R., Wendel D. (2023). Central venous catheter safety in pediatric patients with intestinal failure. Nutr Clin Pract.

[3] Grimaldi C., Gigola F., Bici K., Oreglio C., Coletta R., Morabito A. (2022). Difficult vascular access in children with short bowel syndrome: what to do next?. Children (Basel).

[4] Keefe G., Culbreath K., Staffa S.J., Carey A.N., Jaksic T., Kumar R. (2023). High rate of venous thromboembolism in severe pediatric intestinal failure. J Pediatr.

[5] Rumbo C., Solar H., Ortega M., Busoni V., Barrio S de, Martinuzzi A. (2024). Short bowel syndrome related intestinal failure outcomes in Latin America: insights from the RESTORE Registry. JPEN J Parenter Enteral Nutr.

[6] Neelis E., Koning B de, van Winckel M., Tabbers M., Hill S., Hulst J. (2018). Wide variation in organisation and clinical practice of paediatric intestinal failure teams: an international survey. Clin Nutr.

[7] Schmidt M.L., Wendel D., Horslen S.P., Lane E.R., Brandão L.R., Gottschalk E. (2021). Secondary anticoagulation prophylaxis for catheter-related thrombosis in pediatric intestinal failure: comparison of short- vs long-term treatment protocols. JPEN J Parenter Enteral Nutr.

[8] Monagle P., Chan A.K.C., Goldenberg N.A., Ichord R.N., Journeycake J.M., Nowak-Göttl U. (2012). Antithrombotic therapy in neonates and children: antithrombotic therapy and prevention of thrombosis, 9th ed: American College of Chest Physicians Evidence-Based Clinical Practice Guidelines. Chest.

[9] Wendel D., Mezoff E.A., Raghu V.K., Kinberg S., Soden J., Avitzur Y. (2021). Management of central venous access in children with intestinal failure: a position paper from the NASPGHAN Intestinal Rehabilitation Special Interest Group. J Pediatr Gastroenterol Nutr.

[10] Cohen O., Levy-Mendelovich S., Ageno W. (2020). Rivaroxaban for the treatment of venous thromboembolism in pediatric patients. Expert Rev Cardiovasc Ther.

[11] Hong W.B.-T., Tan W.K., Law L.S.-C., Ong D.E.-H., Lo E.A.-G. (2021). Changes of drug pharmacokinetics in patients with short bowel syndrome: a systematic review. Eur J Drug Metab Pharmacokinet.

[12] Klomberg R.C.W., Vlug L.E., Koning BAE de, Ridder L de (2022). Venous thromboembolic complications in pediatric gastrointestinal diseases: inflammatory bowel disease and intestinal failure. Front Pediatr.

[13] Bosch-Schips M., Artaza G., Hernández-Mata C., Pérez Beltrán V., Cabello Ruiz V., Olivera Sumire P. (2024). Managing venous thrombosis in a pediatric patient with short bowel and congenital nephrotic syndromes: a case report emphasizing rivaroxaban level monitoring. Front Pediatr.

[14] Jaffray J., Young G. (2022). Direct oral anticoagulants for use in paediatrics. Lancet Child Adolesc Health.

[15] European Public Assessment report (2024). Xarelto (rivaroxaban) [medicinal product]: summary of product characteristics. https://www.ema.europa.eu/en/documents/product-information/xarelto-epar-product-information_en.pdf.

[16] van Ommen C.H., Tabbers M.M. (2010). Catheter-related thrombosis in children with intestinal failure and long-term parenteral nutrition: how to treat and to prevent?. Thromb Res.

[17] Young G., Lensing A.W.A., Monagle P., Male C., Thelen K., Willmann S., EINSTEIN-Jr. Phase 3 Investigators (2020). Rivaroxaban for treatment of pediatric venous thromboembolism. An Einstein-Jr phase 3 dose-exposure-response evaluation. J Thromb Haemost.

[18] Lensing A.W.A., Male C., Young G., Kubitza D., Kenet G., Patricia Massicotte M. (2018). Rivaroxaban versus standard anticoagulation for acute venous thromboembolism in childhood. Design of the EINSTEIN-Jr phase III study. Thromb J.

[19] Bosch A., Albisetti M. (2022). Adverse events of DOACs in children. Front Pediatr.

[20] Moran-Lev H., Kocoshis S.A., Oliveira S.B., Helmrath M., Cole C.R. (2023). Chronic mucosal inflammation in pediatric intestinal failure patients—a unique phenomenon. J Pediatr Gastroenterol Nutr.

[21] Furman K., Giustini A., Branstetter J., Woods G., Downey L.A. (2024). A review of the perioperative management of direct oral anticoagulants for pediatric anesthesiologists. Paediatr Anaesth.

